# Electrochemical Generation and Detection of Transient Concentration Gradients in Microfluidic Channels. Theoretical and Experimental Investigations

**DOI:** 10.3389/fchem.2019.00704

**Published:** 2019-10-24

**Authors:** Thomas Abadie, Catherine Sella, Pierre Perrodin, Laurent Thouin

**Affiliations:** PASTEUR, Département de chimie, École normale supérieure, PSL University, Sorbonne Université, CNRS, Paris, France

**Keywords:** microfluidics, electrochemistry, concentration gradient, diffusion, convection, Taylor-Aris

## Abstract

Transient concentration gradients generated and detected electrochemically in continuous flow microchannels were investigated by numerical simulations and amperometric measurements. Operating conditions including device geometry and hydrodynamic regime were theoretically delineated for producing gradients of various profiles with tunable characteristics. Experiments were carried out with microfluidic devices incorporating a dual-channel-electrode configuration. Under these conditions, high electrochemical performance was achieved both to generate concentration gradients and to monitor their dynamics along linear microchannels. Good agreement was observed between simulated and experimental data validating predictions between gradient properties and generation conditions. These results demonstrated the capability of electrochemical microdevices to produce *in situ* tunable concentration gradients with real-time monitoring. This approach is versatile for the active control in microfluidics of microenvironments or chemical gradients with high spatiotemporal resolution.

**Graphical Abstract:**
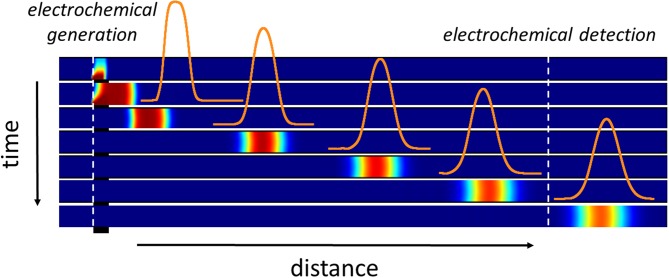
Illustration of the electrochemical generation of a concentration gradient within the microchannel. Variation of the concentration profiles as a function of time and distance from the first working electrode.

## Introduction

In recent years, microfluidic devices have been used in biomolecular and chemical gradient generation with special interest. Spatial and temporal concentration gradients play an important role in many biological assays. They reproduce cellular environments at the microscale and stimulate various cell behaviors such as cell growth, embryogenesis, wound healing, and cancer metastasis (Toh et al., 2013; Somaweera et al., 2016). Chemical gradients are applied to other fields such as chemotaxis (Kim et al., 2010; Mahdavifar et al., 2013), drug design (Chen et al., 2012), and chemical synthesis (Abou-Hassan et al., 2009). They are also relevant for sensor calibration and sample quantification (Wojtowicz et al., 2007). Microfluidics is particularly amenable to gradient generation as length scales in fluid processes are considerably reduced (Whitesides, 2006; Chiu et al., 2017). Apart from processes involving serial dilutions (Wan and Yin, 2018; Jeon et al., 2019), microfluidic systems offer alternative strategies to macroscale methods to achieve tailored gradient profiles (Wang, 2009) with unprecedented spatiotemporal resolutions (Weibel and Whitesides, 2006; Mark and Haeberle, 2010; Velve-Casquillas et al., 2010; Wang et al., 2017). However, the capability of microfluidics to produce tunable concentration gradients with real-time probing is still challenging. Laminar flow based generators are limited to the production of arbitrary profiles (Lee et al., 2009) whose reliability relies heavily on fluid replenishment and accurate flow control. Few studies have focused on the generation of controllable concentration gradients combined with concentration monitoring. In this context, electrochemical reactions in microfluidic systems hold considerable promise both for generation (Mitrovski and Nuzzo, 2005; Klauke et al., 2006; Liu and Abbott, 2011; Contento and Bohn, 2014; Xu et al., 2015) and detection (Contento and Bohn, 2014) of concentration gradients. Indeed, electrochemical techniques are suitable for miniaturization and they are easy to implement in microfluidic devices. Moreover, they provide better sensitivity compared to optical techniques (Gencoglu and Minerick, 2014). In parallel, they can be exploited to develop strategies based on the active control of microenvironments. As examples, some electrochemical approaches have demonstrated the dynamic control of pH in constrained volumes (Fomina et al., 2016; Balakrishnan et al., 2018) or the on-demand oxygen generation (Xu et al., 2015). Applications are immediate in biology but these approaches also open new avenues for the implementation of biochemistry and efficient chemistry. They are not only limited in the production of concentration gradients of electroactive species since non-electroactive species can be also generated by fast and homogeneous chemical reactions coupled to electrode reactions. Despite the potential of electrochemistry for exerting precise regulation or local modulation, the majority of electrochemical platforms are used for passive sensing (Li et al., 2013, 2014, 2018; Oliveira et al., 2013, 2018; Bellagha-Chenchah et al., 2015; Horny et al., 2016; Anderson and Crooks, 2017; Wan and Yin, 2018) rather than for active control.

**Figure 1:**
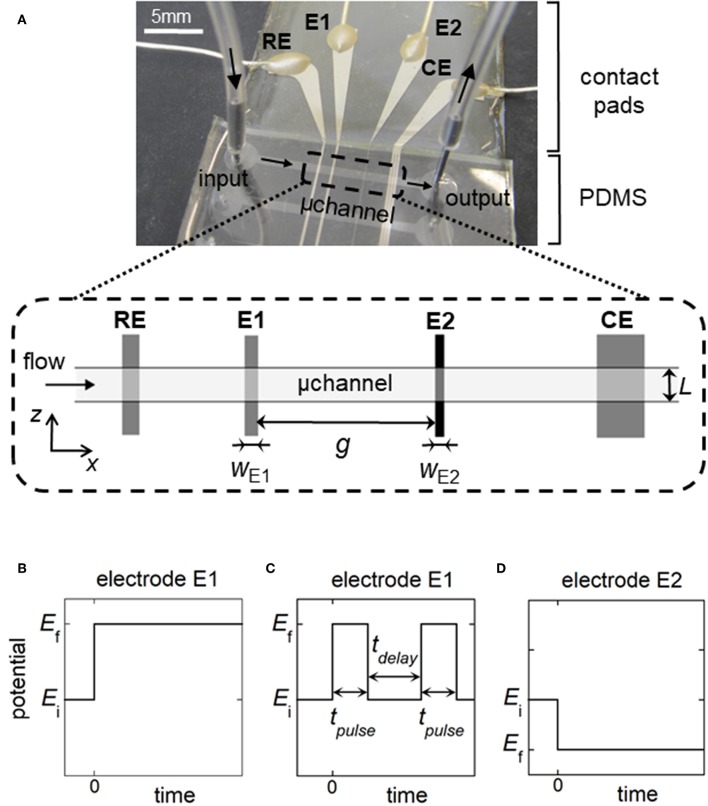
**(A)** Top view of the microfluidic device showing the relative positions of the reference electrode (RE), working electrodes (E1, E2), and counter electrode (CE) within a rectangular microchannel of width *L*. *w*_E1_ and *w*_E2_ are the electrode sizes. *g* is the gap distance between the working electrodes. **(B,C)** Potential pulses for generation of concentration gradients at E1: single step of potential for fronts **(B)**, double step of duration *t*_pulse_ for generation of plugs or peaks **(C)**. *t*_delay_ is the time delay between two potential pulses. **(D)** Potential pulse at E2 for the detection of fronts, plugs, or peaks. In **(B–D)**, *E*_i_ is the initial potential and *E*_f_ is the final potential.

In this work, we investigated through a conceptual approach the electrochemical generation and/or monitoring of transient concentration gradients within microchannels (Graphical Abstract). This concept relies on a dual-channel-electrode configuration operating in generator-collector mode under potentiostatic conditions (Figure 1A). Such systems have been exploited to study homogeneous reaction kinetics (Unwin and Compton, 1989; Fisher and Compton, 1991; Unwin, 1991; Bitziou et al., 2013), catalysis (Dumitrescu et al., 2012), corrosion (Itagaki et al., 1997; Sasaki and Maeda, 2010), and reaction products (Wang et al., 2010). They have also been used for surface titration (Anderson et al., 2017), analyte differentiation (Bitziou et al., 2014; Hu and Fritsch, 2016), and flow velocity determination (Amatore et al., 2004). Operating regimes in these systems can be easily tuned to enhance mass transfer between electrodes and to achieve high collection efficiency with fast kinetics. In combination, numerical modeling facilitates the analysis of experimental results by the identification of key parameters (Amatore et al., 2008a; Holm et al., 2019). Nevertheless, investigations on dual channel-electrodes have been mainly conducted for steady-state processes under conditions similar to those experienced at rotating ring-disk electrodes. They do not exploit the dynamic processes initially established between electrodes. At short time scale, transient gradients can be produced at an electrode with concentration amplitude and length that are a function of the operating conditions and residence time along microchannels (Amatore et al., 2011b). In comparison to generation methods based on microfluidic systems, concentration gradients are produced along the microchannel and not transversally. Concentration gradients of different shapes can be generated temporally to allow for example a sequence of various stimulus patterns at a specific location downstream of the generating electrode. Complex fluidic network with multiple inlets are not required as in microfluidic systems where flowing streams are combined in a gradient chamber after a series of splitting and mixing steps. In these cases, the design of the network defines the shape of concentration gradients and the number of branches determines their spatial resolution (Dertinger et al., 2001; Yang et al., 2002; Lin et al., 2004). The number of splitting steps improves also the stability and accuracy of the gradients but it leads in turn to the design of large networks and greater possibilities of blocking or leakage due to high input driving pressure. Finally, shearing is introduced by increasing the speed of gradient generation within the chamber.

The first advantage of the dual-channel-electrode configuration is straightforward. Generation of concentration profiles can be fully controlled at the first electrode leading to a precise and tunable regulation of species produced during the electrochemical reaction. The second advantage is that the electrode located downstream can be employed to monitor the gradients and their dynamics, at a given distance or time. The process involved between electrodes depends on the diffusive and convective regimes encountered in microchannels. In the following, the electrochemical generation of transient concentration gradients was examined theoretically and experimentally in linear microfluidic microchannels. Numerical simulations were carried out to predict gradient profiles with specific and tunable characteristics. Overall operating conditions were investigated according to the device geometry, flow velocity, and potential pulses applied to the electrodes. In parallel, experiments were performed using the dual-channel-electrode configuration to electrochemically generate and detect transient concentration gradients. Comparisons between theoretical and experimental data were established to assess the validity of the predictions.

## Materials and Methods

### Materials and Reagents

Aqueous solutions of 0.5 × 10^−3^ mol L^−1^ ferrocene methanol (97% Acros organics) were prepared in 0.1 mol L^−1^ potassium chloride (99% Fluka) used as supporting electrolyte. Water was preliminary purified by a Milli-Q purification system. Under such conditions, the diffusion coefficient of ferrocene methanol and ferricinium methanol was equal to *D* = 7.6 × 10^−6^ cm^2^ s^−1^ (Amatore et al., 2011a). The standard potential of the redox couple was estimated to 0.1 *V*/RE.

### Electrochemical Platforms

The microfluidic devices consisted of hybrid PDMS-glass chips. Their design and microfabrication were reported in previous works (Amatore et al., 2004, 2007). Linear channels of 1.5 cm length with rectangular sections (height *h* = 20 or 24 μm, width *L* = 510 or 790 μm) were made by casting polydimethylsiloxane (PDMS, RTV-615; Momentive Performance Materials) onto a patterned mold of SU-8 2015 photoresist (Microchem). Inlet and outlet tubes were punched in the PDMS layer. Each device comprised three parallel microchannels. During one experiment, only one microchannel was filled with flowing solutions, the others remaining empty. Platinum microband electrodes (Ti/Pt with 20 nm/40 nm thickness) were patterned on the glass substrate by soft lithography and deposited using a sputtering coater (K675XD; Emitech). The reference electrode (RE) was fabricated by sputtering 50 nm Ag onto the underlying Pt surface. After the lift-off procedure, the PDMS and the glass slide were exposed to air plasma (Harrick) before bonding them together irreversibly. The electrodes being oriented perpendicular to the section of the main channel, the microband lengths were delimited by the channel width *L*. The reference electrode (RE) and counter electrode (CE) were 200 and 600 μm width, respectively. Before use, RE was oxidized by 5 × 10^−3^ mol L^−1^ FeCl_3_ (Sigma) solution. The width of the first working electrode (E1) was 97 or 200 μm. The width of the second working electrode (E2) was 17 or 30 μm. The gap between E1 and E2 ranged from 400 to 2600 μm.

### Electrochemical Experiments

All electrochemical experiments were performed at room temperature using a homemade multipotentiostat adapted from an original design (Maisonhaute et al., 2001). The ferrocene methanol solution was flowing continuously within the microchannel. During electrochemical generation, electrode E1 was biased at *E* = 0.35 V/RE on the oxidation plateau of ferrocene methanol. For electrochemical detection, E2 was biased at *E* = −0.15 V/RE. The amperometric responses of both electrodes were monitored simultaneously. The flow within the microchannel was pressure driven by means of a syringe pump (Harvard Apparatus, type 11 Pico Plus). The average flow velocities were calibrated following a known procedure (Amatore et al., 2004).

### Numerical Simulations

Concentration profiles and current responses of electrodes were numerically evaluated by solving the mass transport equation with appropriate boundary conditions (Amatore et al., 2011b). Flow was considered laminar with a parabolic velocity profile. Since the microchannel width is much larger than the working electrode widths, the diffusional contribution at each end of working electrodes is negligible. The formulation of the problem was thus reduced in a 2D space. COMSOL Multiphysics 5.4 software was used to perform finite element simulations with the introduction of dimensionless parameters.

## Results and Discussion

### Principle

**Figure 2:**
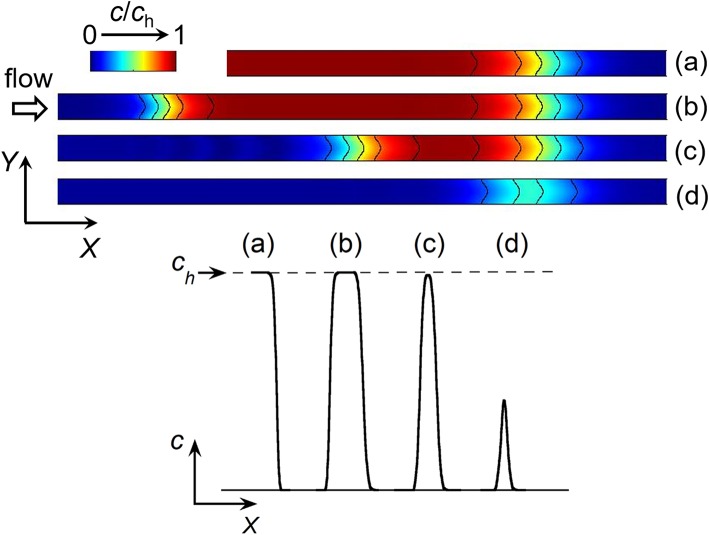
Top: Side view of simulated concentration profiles at a given time between the two working electrodes E1 and E2 in a linear microchannel: front (a), plug (b), and peak (c,d). The black solid curves on the concentration profiles represent isoconcentration lines. Bottom: Concentration variations along *X*-axis illustrating the different types of concentration gradient. *c*_h_ is the maximal concentration reached within a front (a) or plug (b).

The electrochemical cell is based on a four-electrode configuration including two working electrodes. The top view of the device is shown in Figure 1A. All the electrodes are positioned on the microchannel floor. The pseudo-reference electrode is located upstream to ensure its potential stability during the electrochemical cell operation. The counter electrode is situated downstream to not influence the two working electrodes. The first working electrode E1 generates concentration gradients by oxidizing or reducing electroactive species initially present in flowing solution. The second electrode E2 detects downstream the generated gradients by scanning their passage. The two working electrodes are separated by a given gap distance and operate in generator-collector mode. In such a situation, E1 and E2 are biased independently at potentials that ensure in chronoamperometry the control of electrochemical reactions by mass transfer. Two types of potential pulses is applied at E1: a single step or a double step of potential (Figures 1B,C). In both cases, E2 is biased at a constant potential (Figure 1D). As illustrated in Figure 2, different profiles of concentration gradient can be produced according to the potential pulses at E1. In the case of a single step (Figure 1B), the ensuing gradient is a front of concentration that propagates along the microchannel with concentrations ranging from an initial to a maximal level. In the case of a double step of duration *t*_pulse_ (Figure 1C), the gradient is in the form of a concentration pulse having peak shape or plug shape (Figure 2). Peaks are generated at short *t*_pulse_ over small distances. In contrast to peaks, plugs are established at longer *t*_pulse_ over larger distances. They display a plateau with a maximal concentration amplitude. The concentration *c*_h_ reached at maximum for plugs and concentration fronts depends on the average flow velocity and device geometry (i.e., microchannel height and size of E1) (Amatore et al., 2007). *c*_h_ is lower or equal to the concentration *c*_0_ of electroactive species consumed at E1. All parameters including *t*_pulse_ define the operating conditions to tune the gradient profiles at a given location or residence time along the microchannel. Since the concentration gradients are submitted to Taylor-Aris dispersion, they evolve with time (Taylor, 1953; Aris, 1956; Dutta et al., 2006). Hence, the dynamic of the overall process from generation to propagation is controlled by diffusion and convection. Gradient profiles can be monitored downstream by E2 during detection. Indeed, provided that specific conditions are fulfilled, E2 can operate as a concentration probe (Amatore et al., 2011a). In this case, the current response at E2 strictly reflects the concentration variation at the electrode location without any temporal distortion. This property allows establishing the gradient profiles from the current responses.

### Theoretical Predictions for Generating Concentration Gradients

In the following, numerical simulations were performed in order to delineate the conditions for generating each type of concentration gradient as a function of the device geometry and hydrodynamic flow. A laminar regime was considered with a parabolic velocity profile. The width of the microchannel was supposed sufficiently large vs. its height to neglect the influence of walls along the flow direction. A two-dimensional system was thus introduced with dimensionless parameters for:

- coordinates $X=\frac{x}{h}$ and $Y=\frac{y}{h}$ with *h* the microchannel height,
- concentration of generated species $C=\frac{c}{c_{0}}$ with *c*_0_ the initial concentration of species at the entrance of the microchannel,
- size $W_{\text{i}}=\frac{w_{\text{i}}}{h}$,
- distance $G_{\text{i}}=\frac{g_{\text{i}}}{h}$,
- flow velocity $Pe=\frac{u_{\text{av}}h}{D}$ with *Pe* the Peclet number, *u*_av_ the average velocity within the microchannel and *D* the diffusion coefficient of species,
- and time $\tau \;=\frac{Dt}{h^{2}}$.

The origin of coordinates *X* and *Y* is the downstream edge of electrode E1.

#### Generation of Concentration Fronts

**Figure 3:**
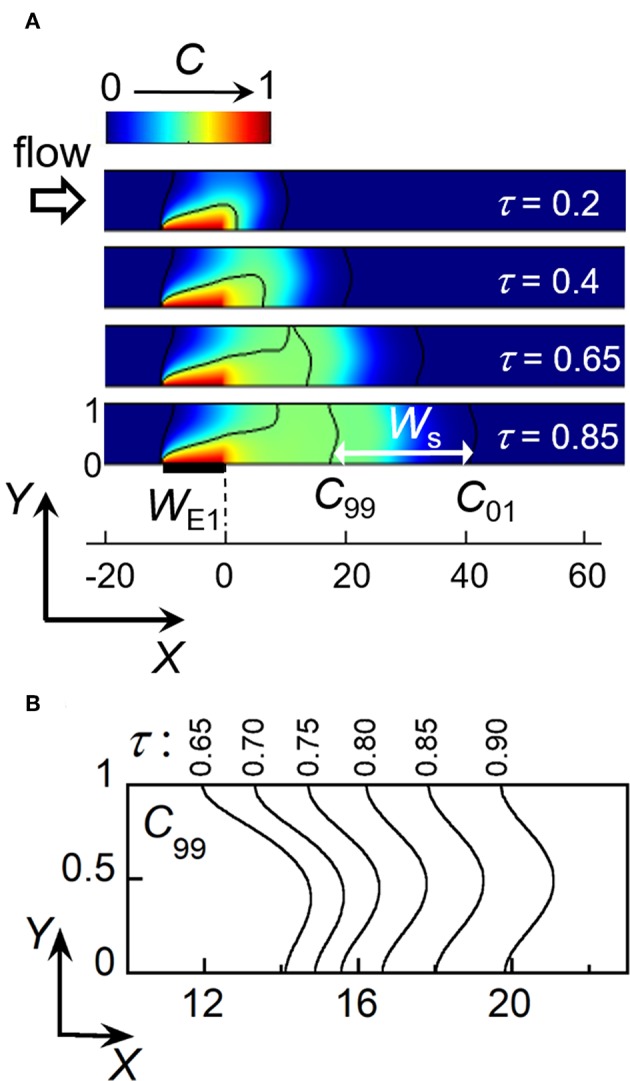
Electrochemical generation of a concentration front and its propagation within a linear microchannel. **(A)** Simulated concentration profiles as a function of time at location close to electrode E1. *W*_s_ is the minimal width between isoconcentration lines *C*_99_ (*C*/*C*_h_ = 0.99) and *C*_01_ (*C*/*C*_h_ = 0.01) at time τ_*s*_ = 0.85. **(B)** Evolution downstream of the isoconcentration line *C*_99_ for times τ between 0.65 and 0.9. In **(A,B)**, *W*_E1_ = 10 and *Pe* = 40.

Simulations were performed under operating conditions corresponding to a single step experiment at E1 (Figure 1B). Conditions were first studied to produce well-established concentration fronts, i.e., displaying parallel isoconcentration lines along the gradient width. Figure 3A shows some simulated concentration profiles as a function of time of species generated at E1. As observed, the amplitude of the concentration front is set by the maximal concentration *C*_h_ reached under these conditions. *C*_h_ is a steady concentration, which depends only on electrode size *W*_E1_ and flow velocity *Pe*. In convective regime and when *W*_E1_/*Pe* < 0.2*, C*_h_ is given by the equation (Amatore et al., 2007):

(1) $$\begin{array}{r} C_{\text{h}}=1.47\;{W_{\text{E}1}}^{2/3}Pe^{-2/3} \end{array}$$

In the following, the front is considered as fully established as soon as two isoconcentration lines characterizing the gradient width become parallel and symmetrical in shape. In Figure 3A, the black lines represent isoconcentration lines monitored for *C/C*_h_ = 0.99 (*C*_99_) and *C/C*_h_ = 0.01 (*C*_01_), respectively. In this case, the criterion is fulfilled when *C*_99_ displays the same steady shape as *C*_01_ located downstream. As shown in Figure 3A, *C*_99_ develops at E1 and then splits into two parts (at τ = 0.65**)**. One is a steady-state isoconcentration line *C*_99_ originating from the upstream edge of E1. The other is the isoconcentration line *C*_99_ corresponding to the propagation of concentration front downstream. This is only after a given residence time τ_s_ that this line *C*_99_ becomes symmetrical and identical in shape to *C*_01_ (Figure 3A, τ_s_ = 0.85). Figure 3B illustrates its evolution at different times for a given electrode size *W*_E1_ and flow velocity *Pe*. τ_s_ can be estimated when both *X* coordinates of *C*_99_, at *Y* = 0 (microchannel floor) and *Y* = 1 (microchannel top), start to coincide. At time τ_s_, the above criterion is met with *G*_s_ the *X* coordinate of *C*_99_ and *W*_s_ the minimal width of concentration front between *C*_99_ and *C*_01_ (see also Figure 3A). Note that contrary to the velocity profile of laminar flow, the resulting isoconcentration lines are not parabolic. Indeed, close to the walls (at *Y* = 0 and *Y* = 1) the flow velocity is low, leading to the prevalence of transversal diffusion and subsequent adjustment of concentration profile from the parabolic one (Amatore et al., 2008b).

**Figure 4:**
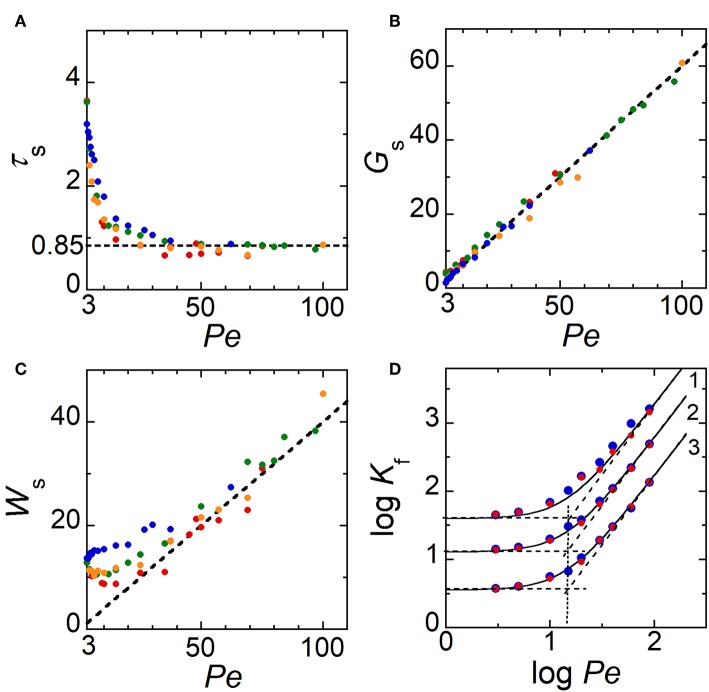
**(A–C)** Theoretical variations of τ_s_, *G*_s_, and *W*_s_ as a function of *Pe* for several electrode sizes *W*_E1_. The dashed lines correspond to τ_s_ = 0.85, *G*_s_ = 0.6 *Pe*, and *W*_s_ = 0.4 *Pe*, respectively. **(D)** Variation of log *K*_f_ as a function of log *Pe* for three couples of isoconcentration lines: 1 (*C*_99_, *C*_01_); 2 (*C*_90_, *C*_10_), and 3 (*C*_75_, *C*_25_). The solid lines correspond to Equation (5) when γ_f_ = 40 (*C*_99_, *C*_01_), γ_f_ = 13 (*C*_90_, *C*_10_), and γ_f_ = 3.6 (*C*_75_, *C*_25_). Dashed lines represent the two limiting cases. In **(A–C)**, symbols correspond to data evaluated from numerical simulations with *W*_E1_ = 9.85 (blue), 4.85 (orange), 2.85 (green), and 0.85 (red). In **(D)**, *W*_E1_ = 10 (blue) and 1 (red).

Figure 4 reports variations obtained from simulations of τ_s_, *G*_s_ and *W*_s_ as a function of *Pe*, for several sizes *W*_E1_ of electrode E1 ranging from 0.8 to 10. In convective regimes, when *Pe* > 30, data show that τ_s_ does not depend on *W*_E1_. τ_s_ is almost constant with an average value close to 0.85 (Figure 4A). Distance *G*_s_ varies linearly with *Pe* (Figure 4B) according to the relation:

(2) $$\begin{array}{r} G_{\text{s}}=0.6Pe \end{array}$$

The factor 0.6 corresponds here to the average time *G*_s_/*Pe* required by *C*_99_ to reach its steady shape at distance *G*_s_ from the downstream edge of E1. This time is lower than τ_s_ since it does not include the time delay needed for *C*_99_ to develop over the electrode surface. At τ_s_, *W*_s_ is almost independent on *W*_E1_ (Figure 4C) but depends strongly on *Pe*. When *Pe* > 30, a linear relation is clearly noticed with:

(3) $$\begin{array}{r} W_{\text{s}}=0.4Pe \end{array}$$

Indeed, the generation of concentration front is fully controlled by diffusion and convection. After being established at time τ_s_, concentration gradients propagate under the influence of hydrodynamic dispersion given by Taylor-Aris theory. If *W*_f_ is the width of concentration front at times higher than τ_s_, *W*_f_ necessarily follows the relation for solute dispersion under pressure-driven flow with (Dutta et al., 2006):

(4) $$\begin{array}{r} W_{\text{f}}=\sqrt{K_{\text{f}}\tau } \end{array}$$

(5) $$\begin{array}{r} \text{and }K_{\text{f}}=\gamma_{\text{f}}\left(1+\frac{Pe^{2}}{210}\right) \end{array}$$

γ_f_ is a constant whose value depends on *W*_f_, i.e., on the couple of isoconcentration lines considered to characterize *W*_f_. Simulations were thus performed at times higher than τ_s_ to estimate γ_f_ from Equations (4) and (5). Figure 4D shows *K*_f_ data evaluated from *W*_f_ with three couples of isoconcentration lines (*C*_99_, *C*_01_), (*C*_90_, *C*_10_), and (*C*_75_, *C*_25_). As expected in Equation (5), two limiting behaviors are obtained according to *Pe* with transitions occurring close to the critical value $Pe=\sqrt{210}$ ~ 15. By fitting *K*_f_ variations with Equation (5), the constant γ_f_ was estimated to γ_f_ = 40 for (*C*_99_, *C*_01_), γ_f_ = 13 for (*C*_90_, *C*_10_), and γ_f_ = 3.6 for (*C*_75_, *C*_25_). Data showed also that when *Pe* > 30, Equation (4) was equivalent to Equation (3) for (*C*_99_, *C*_01_) with γ_f_ = 40 Under these conditions, it can be assumed that at τ_s_ concentration fronts are controlled by Taylor-Aris dispersion.

Therefore, Equations (1)–(5) and γ_f_ values are useful for predicting the positions of the generated concentration fronts, their concentration amplitude *C*_h_ and width *W*_f_ along the microchannel, according to flow velocity *Pe* and time. These predictions are independent of the range of electrode width *W*_E1_ investigated. Note also that under these operating conditions, the duration of E1 polarization allows establishing and controlling within the microchannel a zone of homogeneous concentration *C*_h_ downstream of E1.

#### Generation of Concentration Pulses

**Figure 5:**
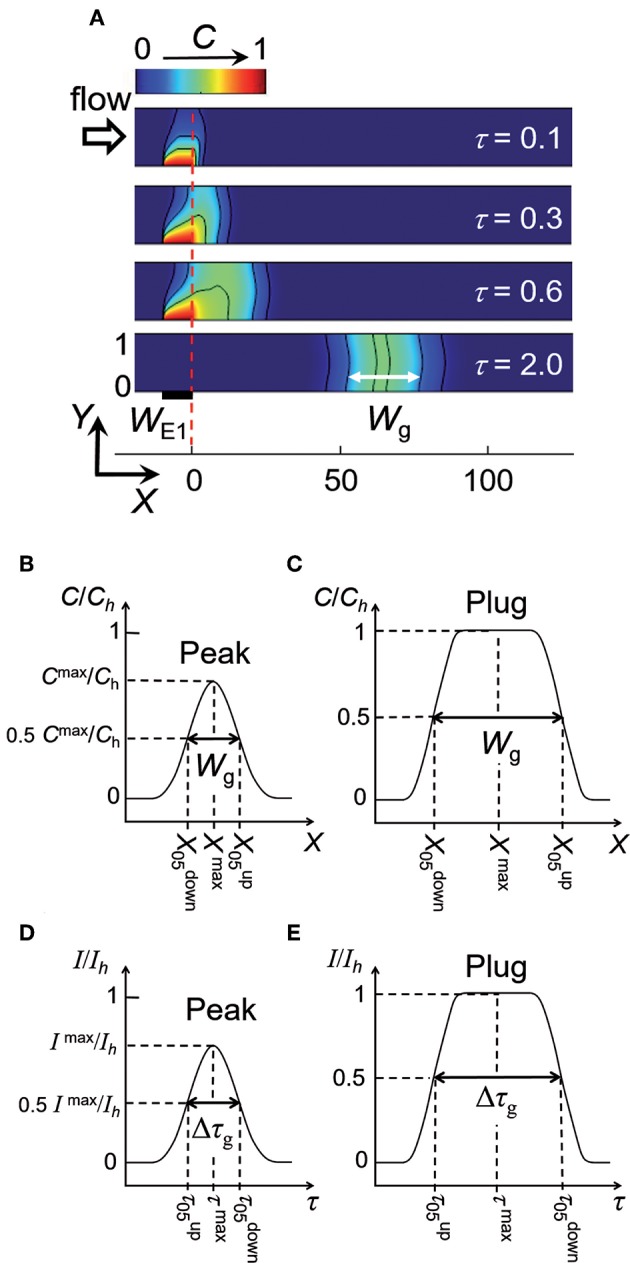
**(A)** Electrochemical generation of a concentration peak and its propagation within a linear microchannel. Concentration profiles are reported at different times τ. *W*_E1_ = 10, *Pe* = 40 and τ_pulse_ = 0.6. *W*_g_ is the width defined between the two isoconcentration lines *C*_50_. **(B,C)** Characteristic parameters of peaks and plugs deduced from concentration variations along *X*-axis. **(D,E)** Characteristic parameters of peaks and plugs deduced from current responses monitored at electrode E2. *I*_h_ is the maximal current reached for fronts or plugs.

**Figure 6:**
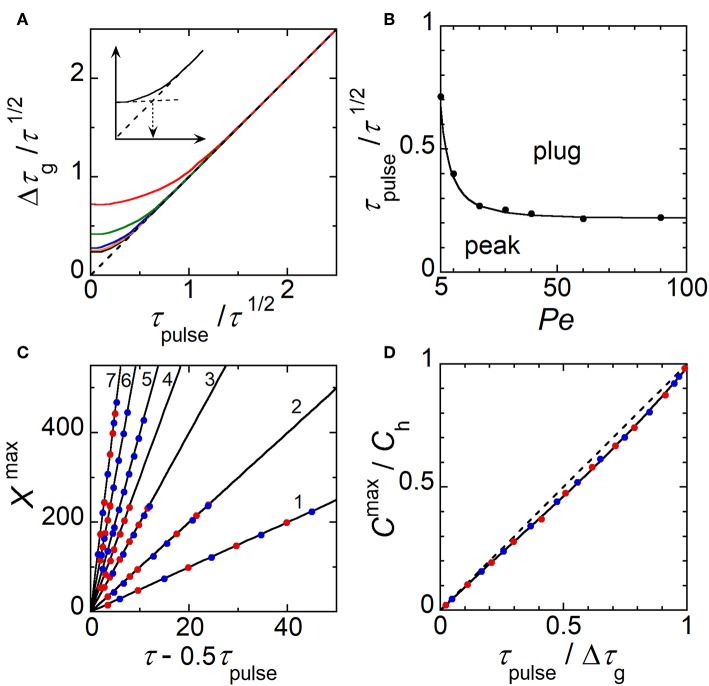
**(A)** Variations of Δτ_g_/τ ^1/2^ as a function of τ_pulse_/τ^½^ for various *Pe*: *Pe* ≥ 40 (black), *Pe* = 30 (orange), *Pe* = 20 (blue), *Pe* = 10 (green), and *Pe* = 5 (red). *W*_E1_ ranged from 1 to 10. The dashed line represents the limiting behavior Δτ_g_/τ ^1/2^ = τ_pulse_/τ^½^. Insert shows extrapolation from the two limiting behaviors. **(B)** Zone diagram (*Pe*, τ_pulse_/τ^½^) giving the operating conditions for generating peaks and plugs, respectively. The solid line corresponds to Equation (9) with γ_g_ = 10. Symbols correspond to evaluations from simulations. **(C)** Variation of *X*^max^ as a function of τ – 0.5τ_pulse_ for *Pe* = 5 (1), 10 (2), 20 (3), 30 (4), 40 (5), 60 (6), and 90 (7). **(D)** Variation of *C*^max^/*C*_h_ as a function of τ_pulse_/Δτ_g_. The dashed line is the equation *C*^max^/*C*_h_ = τ_pulse_/Δτ_g_. In **(C,D)**, *W*_E1_ = 10 (blue) and 1 (red).

Simulations were performed under operating conditions corresponding to a double step of potential at E1 (Figure 1C). As an example, Figure 5A illustrates the generation of a concentration pulse as a function of time. Concentration pulses are well-established with symmetric shape after a time delay necessarily higher than τ_s_. According to τ_pulse_ and *Pe*, concentration pulses can be observed under the form of peaks or plugs (Figures 5B,C). Each type of concentration gradient is characterized by a position *X*^max^, concentration amplitude *C*^max^/*C*_h_, and width *W*_g_ at half height. Note that *C*_h_ is given by Equation (1) and *C*^max^/*C*_h_ is equal to 1 for plugs only. Since plugs transform to peaks with time due to hydrodynamic dispersion, the objective was to examine the operating conditions governing their production (τ_pulse_ and *Pe*) and characteristics (*X*^max^*, C*^max^/*C*_h_, and *W*_g_). *W*_g_ is defined here as the width of concentration pulse. Since Taylor-Aris dispersion operates as a function of τ^1/2^ (see Equation 4), the ratio τ_pulse_/τ^1/2^ was selected as the parameter to account for the operating conditions. In parallel, the ratio Δτ_g_/τ^1/2^ is the parameter that was chosen for characterizing the concentration gradients with Δτ_g_ = *W*_g_/*Pe*. Figure 6A gives the variations of Δτ_g_/τ^1/2^ as a function of τ_pulse_/τ^1/2^ for various electrode sizes *W*_E1_ and velocity *Pe*. At each *Pe*, a same trend was noticed whatever *W*_E1_. Indeed, at low τ_pulse_/τ^1/2^ corresponding to peak generation, Δτ_g_/τ^1/2^ is constant and independent of τ_pulse_/τ^1/2^. In this case, the influence Taylor-Aris dispersion on the concentration gradient is maximal. Conversely, for higher τ_pulse_/τ^1/2^ corresponding to plug generation, Δτ_g_/τ^1/2^ varies linearly with τ_pulse_/τ^1/2^ with a slope equal to 1, evidencing the equality between Δτ_g_ and τ_pulse_. In this case, Taylor-Aris dispersion occurs but is negligible with respect to the large width of plugs. Hence, it is possible from Figure 6A to delineate the conditions leading to the production of peaks or plugs. In Figure 6A (insert), a threshold value of τ_pulse_/τ^1/2^ was evaluated at each *Pe* by extrapolating the two limiting behaviors. This investigation is resumed in Figure 6B by plotting data in a zone diagram (*Pe*, τ_pulse_/τ^1/2^) in order to delimit conditions for observing either peaks or plugs. In this zone diagram, the upper area corresponds to plugs while the lower area stands for peaks. It is possible to derive the equation of the boundary in Figure 6B by considering the influence of Taylor-Aris dispersion on *W*_g_ (Figures 5B,C) with:

(6) $$\begin{array}{r} W_{\text{g}}=\sqrt{K_{\text{g}}\tau } \end{array}$$

(7) $$\begin{array}{r} \text{and }K_{\text{g}}=\gamma_{\text{g}}\left(1+\frac{Pe^{2}}{210}\right) \end{array}$$

γ_g_ is the constant defined for concentration pulses.

Indeed, the boundary corresponds in Figure 6A to the following equality:

(8) $$\begin{array}{r} \frac{\tau_{\text{pulse}}}{\sqrt{\tau }}=\frac{W_{\text{g}}}{Pe\sqrt{\tau }} \end{array}$$

By combining Equations (6) and (8), it leads to:

(9) $$\begin{array}{r} \frac{\tau_{\text{pulse}}}{\sqrt{\tau }}=\frac{\sqrt{K_{\text{g}}}}{Pe} \end{array}$$

In order to check Equation (9) and to assess γ_g_, a fit of the boundary in Figure 6B was performed. In this case, γ_g_ was found equal to 10. Therefore, at low τ_pulse_/τ^1/2^ corresponding to peak generation (lower area of the zone diagram), *W*_g_ can be evaluated from Equations (6) and (7). At high τ_pulse_/τ^1/2^ corresponding to plug generation (upper area of the zone diagram), *W*_g_ is easily deduced by:

(10) $$\begin{array}{r} W_{\text{g}}=Pe\;\tau_{\text{pulse}} \end{array}$$

At first glance, the time required for observing a concentration pulse at the position *X*^max^ can be estimated from the velocity *Pe* and the time difference (τ – 0.5τ_pulse_). Indeed, one expects for plugs the relation:

(11) $$\begin{array}{r} X^{\text{max}}=Pe\left(\tau -0.5\;\tau_{\text{pulse}}\right) \end{array}$$

In Figure 6C, the simulated data show that *X*^max^ varies linearly with (τ – 0.5τ_pulse_) whatever τ_pulse_ and *W*_E1_. The slope is close to *Pe* demonstrating the validity of Equation (11), not only for plugs but also for peaks. Indeed, for peaks *X*^max^ tends to *Pe*τ as τ_pulse_ tends to zero.

The maximal concentration amplitude *C*^max^/*C*_h_, which is a property of peaks, is approximatively equal to τ_pulse_/Δτ_g_ as noticed in Figure 6D. Note that for plugs *C*^max^ equals to *C*_h_ by definition, i.e., *C*^max^/*C*_h_ = 1.

The zone diagram in Figure 6B depicts in dimensionless parameters an infinity of operating conditions to generate electrochemically concentration gradients under the form of peak or plug. According to the electrode size *W*_E1_ and duration τ_pulse_, their characteristics (i.e., shape, position *X*^max^, width *W*_g_, and amplitude *C*^max^) can be modulated at a given distance *X* or residence time τ along the microchannel. Conditions and resulting properties of gradients that were delineated are summarized in Table 1.

**Table 1:** Operating conditions and characteristics of concentration gradients generated under the form of peaks and plugs.

	**Operating conditions**	***W*_g_**	***X*^max^**	***C*^max^/*C*_h_**
Peak	$\frac{\tau_{\text{pulse}}}{\sqrt{\tau }}<<\frac{\sqrt{K_{g}}}{Pe}$	Equations (6) and (7) with γ_g_ = 10	Equation (11)	*C*^max^/*C*_h_ ~ τ_pulse_/Δτ_g_
Plug	$\frac{\tau_{\text{pulse}}}{\sqrt{\tau }}>>\frac{\sqrt{K_{g}}}{Pe}$	Equation (10)	Equation (11)	C^max^ = C_h_

### Detection of Concentration Gradients

The above predictions were checked experimentally by generating and detecting electrochemically some concentration gradients using a dual-working electrode configuration (Figure 1A). The principle of the detection relies on the fact that the second electrode E2 scans the passage of the concentration gradient over its surface as a function of time. In order to characterize accurately the gradient profile, the electrode must probe the concentration variation without any time delay or kinetic distortion. In a previous study, we demonstrated that some operating conditions at microchannel electrodes fulfill such a criterion (Amatore et al., 2011a). Indeed, when *Pe* > 15 in convective regime, the size of electrode E2 must be lower than the critical size ${\text{W}}_{\text{E}2}^{\text{max}}$ given by:

(12) $$\begin{array}{r} W_{\text{E2}}^{\text{max}}=0.49+0.05Pe \end{array}$$

**Figure 7:**
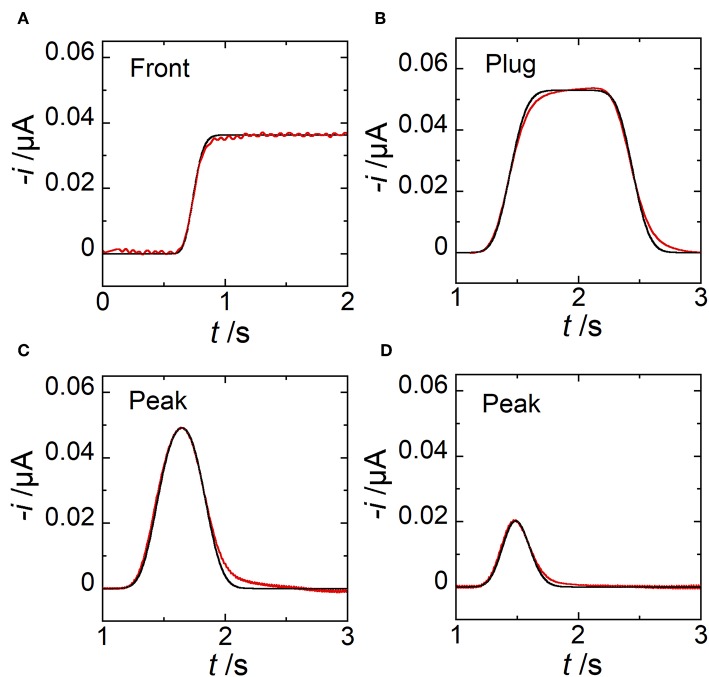
Comparison between simulated (black lines) and experimental (red lines) current responses monitored at electrode E2. **(A)** Case of a concentration front. **(B)** Case of a concentration plug with *t*_pulse_ = 1 s. Estimated *w*_g_ = 1860 μm. **(C)** Case of a concentration peak with *t*_pulse_ = 0.4 s. Estimated *w*_g_ = 744 μm. **(D)** Case of a concentration peak with *t*_pulse_ = 0.1 s. Estimated *w*_g_ = 446 μm. In **(A)** *w*_E1_ = 97 μm, *w*_E2_ = 17 μm, *g* = 1000 μm, *h* = 20 μm, *l* = 510 μm, *u*_av_ = 0.86 μL min^−1^, and *c*_0_ = 0.63 mM. In **(B–D)** *w*_E1_ = 200 μm, *w*_E2_ = 30 μm, *g* = 2600 μm, *h* = 24 μm, *l* = 790 μm, *u*_av_ = 2.12 μL min^−1^, and *c*_0_ = 0.38 mM. In **(A–D)**, each curve is related to a single experiment.

Under these conditions, the electrode response in chronoamperometry strictly follows the concentration variation at the upstream edge of E2. Hence, experimental conditions were selected to produce and detect concentration fronts (Figure 7A), plugs (Figure 7B), and peaks (Figures 7C,D). The distance *G* separating E1 and E2 was sufficiently large to establish concentration gradients with symmetric profiles according to the experimental flow velocities used. In Figures 7A–D are reported the experimental and simulated current responses. The comparison between data led to a very good agreement showing the remarkable accuracy achieved both in predictions and in measurements. Under these conditions, the width of concentration pulses ranged between 446 and 1860 μm. Videos and Figures showing simulated data during the generation, propagation, and detection of a plug (Video S1, Figure S1) and a peak (Video S2, Figure S2) are provided in Supplementary Materials.

**Figure 8:**
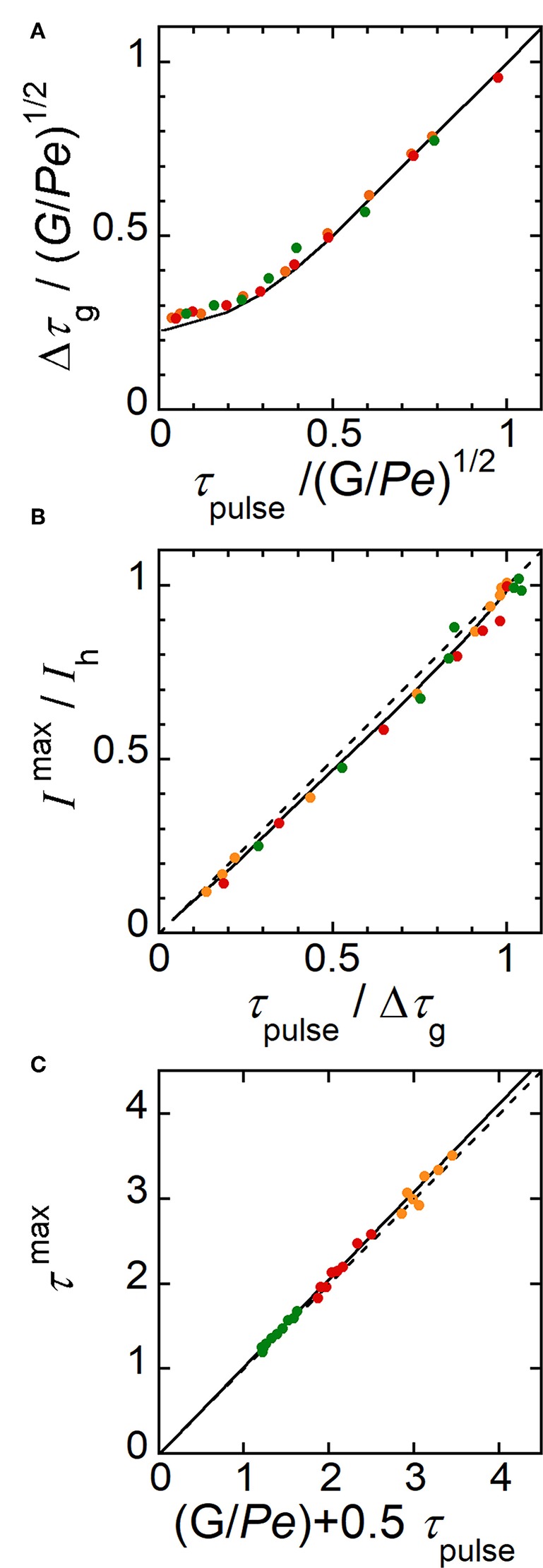
Characteristic parameters evaluated theoretically (solid lines) and experimentally (symbols) from current responses monitored at electrode E2. **(A)** Plot of Δτ_g_/(*G*/*Pe*)^1/2^ as a function of τ_pulse_/(*G*/*Pe*)^1/2^. **(B)** Plot of *I*^max^/*I*_h_ as a function of τ_pulse_/Δτ_g_. The dashed line is the equation *I*^max^/*I*_h_ = τ_pulse_/Δτ_g_. **(C)** Plot of τ_max_ as a function of (*G*/*Pe* + 0.5τ_pulse_). The dashed line is the equation τ_max_ = *G*/*Pe* + 0.5τ_pulse_. In **(A–C)**, experimental flow rate *u*_av_ = 1.40 μL min^−1^ (green), 2.12 μL min^−1^ (red), and 3.27 μL min^−1^ (orange). *w*_E1_ = 200 μm, *w*_E2_ = 30 μm, *g* = 2600 μm, *h* = 24 μm, *l* = 790 μm, and *c*_0_ = 0.38 mM. *t*_pulse_ ranged from 0.1 to 1 s. In **(A–C)**, each data is related to a single experiment.

Extended investigations were thus performed by analyzing the profiles of electrode response (Figures 5D,E) as it was previously realized for concentration profiles (Figures 5B,C). Indeed, like in Figure 6, variations can be established from current responses provided that the concentration gradients do not evolve significantly during their complete detection (i.e., during the time required by E2 to scan the width *W*_g_ of concentration gradient). According to the range of conditions investigated below, the ratio Δ*W*_g_/*W*_g_ did not exceed 0.1 with Δ*W*_g_ the variation of *W*_g_ estimated during detection. Other parameters were considered by introducing the average time *G*/*Pe* and the duration Δτ_g_ of current curves at half height. Current was normalized by the maximal current *I*_h_ reached during plug detection and a corresponding time τ^max^ was defined accordingly (Figures 5D,E). Therefore, similar variations were established from simulated current responses (Figure 8). By plotting Δτ_g_/(*G*/*Pe*)^1/2^ as a function of τ_pulse_/(*G*/*Pe*)^1/2^, a same trend was observed whatever *W*_E1_ at *Pe* higher than 30 (compare Figure 8A with Figure 6A)_._ Similarly, variation of *I*^max^/*I*_h_ as a function of τ_pulse_/Δτ_g_ was almost linear (Figure 8B). Finally, as for Equation (11) a relationship could be derived between τ^max^ and *G*/*Pe* with:

(13) $$\begin{array}{r} \tau^{\text{max}}=\frac{G}{Pe}+0.5\;\tau_{\text{pulse}} \end{array}$$

Corresponding data are reported in Figure 8C. Note that this equation is relevant since it provides a mean to estimate with accuracy the average flow velocity inside microchannel. Working curves established in Figure 8 are complementary tools to those given in Figure 6 since they allow performing similar predictions based here on the monitoring of concentration gradients at E2. These variations were assessed experimentally under wide operating conditions leading to the generation of various gradient profiles. According to the experimental conditions, the width *w*_g_ of concentration pulses ranged from 430 to 1870 μm while *t*_max_ ranged from 0.75 to 3 s. In Figures 8A–C are plotted together experimental (symbols) and simulated current responses (lines). A perfect agreement was noticed for every characteristics Δτ_g_, *I*^max^/*I*_h_ and τ^max^ of the current responses. Therefore, these results validated the underlying concept, which associates the electrochemical generation and the monitoring of transient concentration gradients. Tunable gradients can be predicted from Table 1 and be produced experimentally under extended operating conditions.

**Figure 9:**
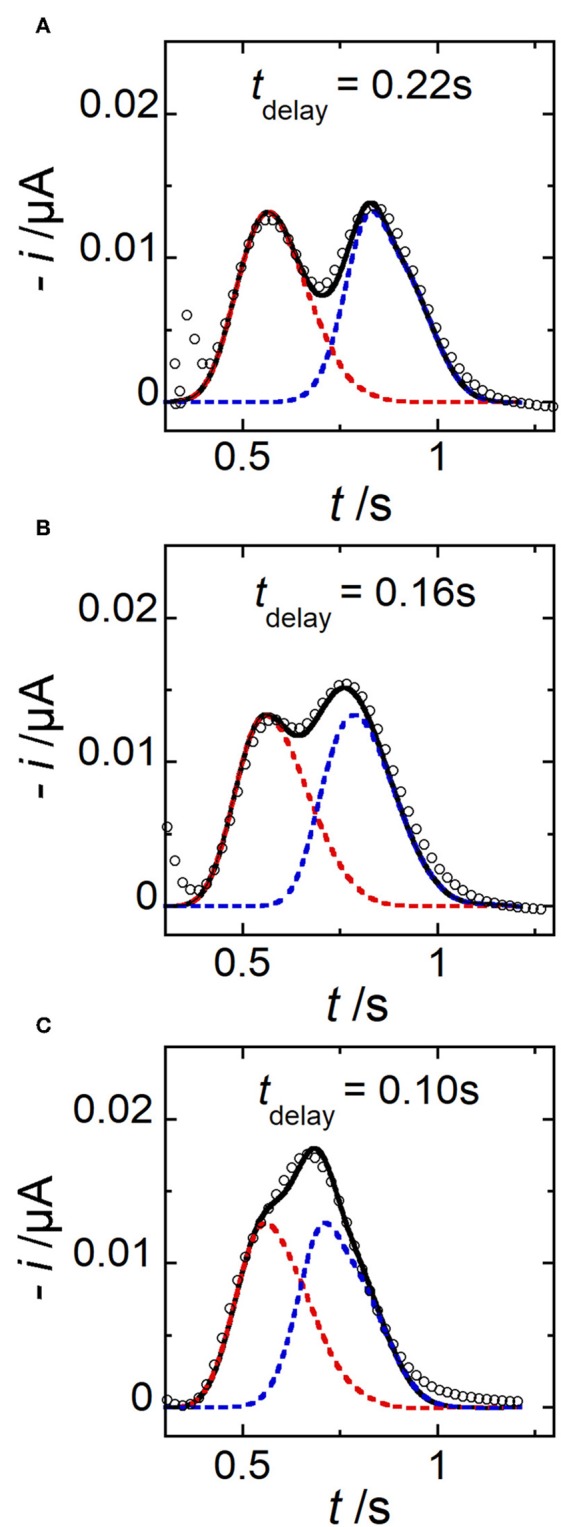
Comparison between simulated (lines) and experimental (symbols) current responses monitored at electrode E2 in the case of two consecutive peaks generated for three different *t*_delay_. **(A)** *t*_delay_ = 0.22 s. **(B)** *t*_delay_ = 0.16 s. **(C)** *t*_delay_ = 0.10 s. In **(A–C)**, *t*_pulse_ = 0.06 s, *w*_E1_ = 200 μm, *w*_E2_ = 30 μm, *g* = 400 μm, *h* = 20 μm, *l* = 790 μm, c_0_ = 0.33 mM, and *u*_av_ = 1 μL min^−1^. Each simulated peak is plotted in dashed line (red or blue). The solid line corresponds to the sum of peak contributions. Each current response is related to a single experiment.

The commutation between different types of concentration gradient can be very fast. To demonstrate further the electrochemical performance achieved under these conditions, extra experiments were carried out with two consecutive potential pulses at electrode E1 (Figure 1C). Two concentration peaks were generated within the microchannel by imposing relatively short time delay *t*_delay_ vs. *t*_pulse_. In this case, peaks may partially overlap or even merge into one single peak. Three different operating conditions were investigated in Figure 9. For comparison, experimental (symbols) and simulated (lines) current responses were reported together. As expected, two peaks were produced and monitored successively. Under these conditions, by decreasing to some extent the time delay *t*_delay_, peaks partially merged, producing intricate patterns. In this situation, the benefit of simulations was to decipher graphically the individual peak contributions from the overall response. As shown in Figure 9, a good agreement was obtained between data, demonstrating the performance in predicting, and generating potentially complex profiles of concentration gradients. In particular, these examples evidenced the high spatiotemporal resolution achieved experimentally that enables to tune and monitor concentration gradients under conditions close to peak convolution.

## Conclusion

A dual-working-electrode configuration was considered to generate and detect by amperometry transient concentration gradients in linear microchannels. Gradients can be produced with adjustable profiles depending on the flow velocity and the duration of potential pulse at a generator electrode. A zone diagram delineating all the operating conditions was established to accurately predict the resulting properties of concentration gradients. In parallel, experiments evidenced the high spatiotemporal resolution achieved for monitoring electrochemically dynamic concentrations. Predictions from numerical simulations associated with experimental validations showed thus the electrochemical performance for generating and controlling *in situ* concentration gradients. They also demonstrated the underlying concept that combines the generation of tunable concentration gradients in microfluidic channels with real-time monitoring. In comparison to generation methods based on microfluidic systems, complex fluidic networks are not required. The concentration gradients are produced along the microchannel, which allows various and consecutive stimulus patterns to be generated downstream. This investigation will certainly benefit a large number of bioanalytical applications for which physicochemical processes are induced locally by highly resolved concentration gradients.

## Data Availability Statement

The datasets generated for this study are available on request to the corresponding author.

## Author Contributions

TA and PP were early stage researchers. They contributed to the experimental measurements. CS performed the simulations and contributed with LT to data analysis. LT proposed the study. CS and LT contributed to the writing of the paper.

### Conflict of Interest

The authors declare that the research was conducted in the absence of any commercial or financial relationships that could be construed as a potential conflict of interest.
