# Supplementary material for: Electrochemical Generation and Detection of Transient Concentration Gradients in Microfluidic Channels. Theoretical and Experimental Investigations
Source: Front Chem. 2019 Oct 24;7:704. doi: 10.3389/fchem.2019.00704 (PMC6822297; doi:10.3389/fchem.2019.00704)
Supplement: Supplementary file 3 [file Data_Sheet_1.PDF]

Supplementary Materials

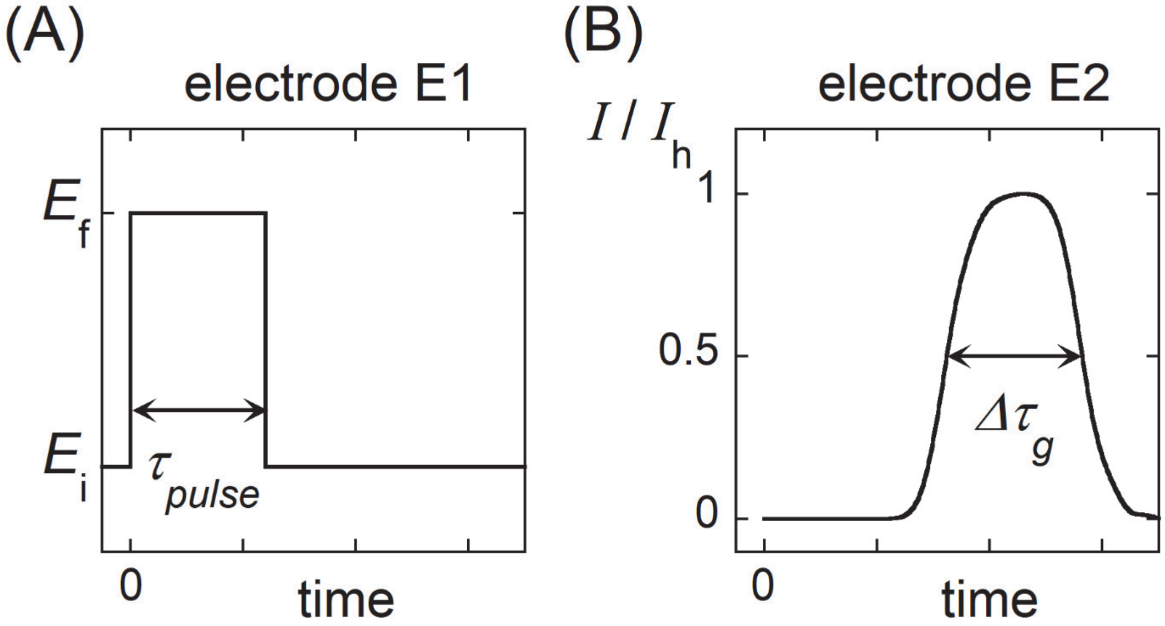

Figure S1: Electrochemical generation and detection of a concentration plug. (A) Potential pulse at E1. (B) Current response monitored at E2. I_h is the maximal current. $W_{E1} = 10$, $W_{E2} = 0.01$, $G = 30$, $Pe = 10$, $\tau_{pulse} = \Delta\tau_g = 2.4$. Data corresponding to **Video S1**.

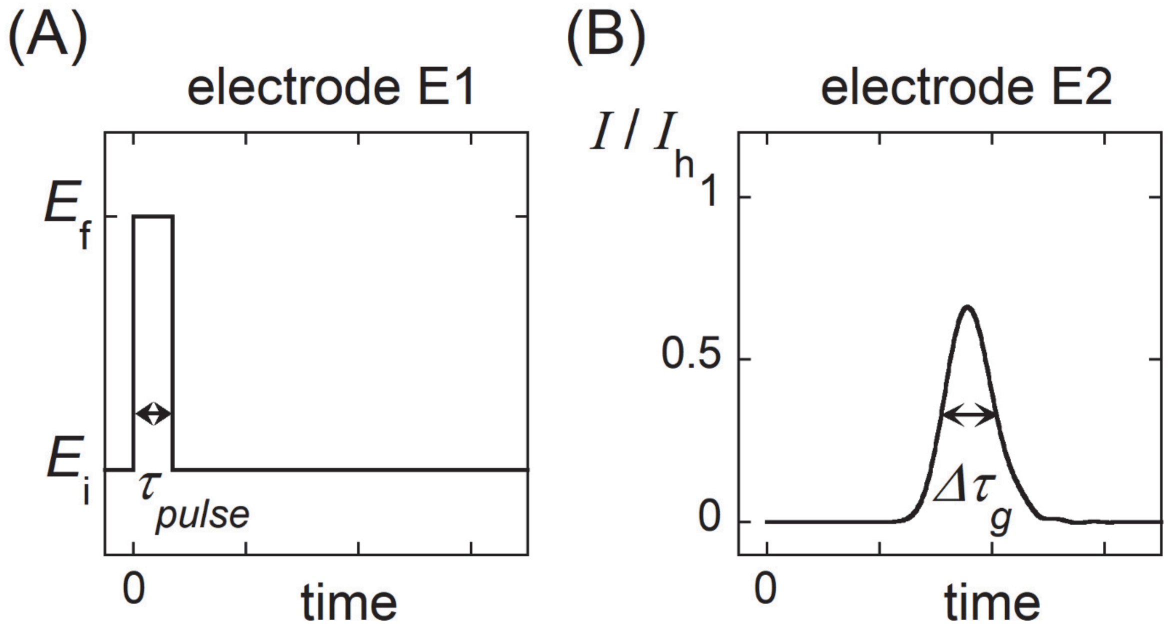

Figure S2: Electrochemical generation and detection of a concentration peak. ((A) Potential pulse at E1. (B) Current response monitored at E2. I_h is the maximal current of a plug. $W_{E1} = 10$, $W_{E2} = 0.01$, $G = 30$, $Pe = 10$, $\tau_{pulse} = 0.7$, $\Delta\tau_g = 0.96$. Data corresponding to **Video S2**.
